# Quality of osteoporosis management guidelines produced in China: a systematic review

**DOI:** 10.3389/fendo.2026.1907308

**Published:** 2026-10-15

**Authors:** Xiaonan Liu, Mengya Ge, Caie Wu, Kai Shen, Jin Yang, Shunxia Sun

**Affiliations:** 1 Department of Anesthesiology, The First Affiliated Hospital of Chongqing Medical University, Chongqing, China; 2 Public Health Department, Chongqing University Three Gorges Hospital, Chongqing, China; 3 Department of Spine Surgery, Chongqing General Hospital, Chongqing, China; 4 Department of Critical Care Medicine, Chongqing General Hospital, Chongqing, China

**Keywords:** AGREE TWO, AGREE-REX, guideline, management, osteoporosis

## Abstract

**Introduction:**

This study employed the AGREE II and AGREE-REX instruments to assess the methodological rigor and recommendation quality of Chinese clinical practice guidelines (CPGs) for osteoporosis care.

**Methods:**

A systematic search was conducted across CNKI, SinoMed, Wanfang, VIP, Medlive, and the Chinese Medical Association website for CPGs published between January 2014 and December 2025. AGREE II was used to evaluate methodological quality, while AGREE-REX was applied to guidelines scoring ≥50% in at least three AGREE II domains.

**Results:**

A total of 13 CPGs were analyzed. The median overall quality score was 54% (IQR: 49%–71%). The rigor of development domain scored the lowest [38% (25%–94%)], whereas stakeholder involvement and clarity of presentation scored the highest [72% (44%–72%) and 72% (61%–83%), respectively]. Ten guidelines met the threshold for AGREE-REX assessment, with a median recommendation quality score of 56% (32%–63%). A positive correlation was found between AGREE-REX and AGREE II overall scores (r = 0.665).

**Conclusions:**

The methodological and recommendation quality of Chinese osteoporosis CPGs is moderate. Recommendations focusing on functional assessment, diet and exercise, rehabilitation, and environmental modifications are valuable. Furthermore, traditional Chinese medicine exercises may offer a reference for CPG development in other countries.

**Systematic review registration:**

https://ebn.nursing.fudan.edu.cn/myRegisterList, identifier, ER20240722.

## Introduction

Osteoporosis is a systemic skeletal disease characterized by reduced bone density and structural deterioration, leading to increased fragility and fracture risk (1, 2). Early-stage primary osteoporosis often presents no obvious symptoms. As bone mass continues to decline, patients may experience pain, spinal deformities, and in severe cases, vertebral or hip fractures (3, 4). This condition poses a significant threat to the health and quality of life of older adults worldwide. According to China’s 2021 national census, the population over 60 years old exceeded 264 million (18.7% of the total), and those over 65 years surpassed 190 million (13.5%), representing the largest elderly population globally (5). The China Development Research Foundation projects that population aging will peak between 2035 and 2050, with those aged ≥65 years reaching 380 million (approximately 30% of the total population) by 2050 (6). Consequently, the incidence of osteoporosis among the elderly is rising annually (7, 8).

Global estimates suggest that by 2050, around 6.3 million hip fractures will occur in older adults, more than half of which will happen in Asia (9). In 2018, the prevalence of osteoporosis in China was 24%, rising to over 60% among those aged ≥65 years. More than one million new hip fractures are recorded each year in china, and this figure continues to grow (10).

Osteoporosis-related guidelines play a crucial role in guiding diagnosis, treatment, and disease management. In 2003, the Scottish Intercollegiate Guidelines Network (SIGN) issued clinical guidelines for osteoporosis. Subsequently, organizations such as the American College of Physicians, the Endocrine Society, the National Osteoporosis Guideline Group (NOGG), and the National Osteoporosis Foundation have published their own guidelines (11–14). In China, the first guidelines for osteoporotic fractures were released in 2008 by the Chinese Academy of Osteology (15), followed by the first primary osteoporosis guidelines in 2011 from the Academy’s Osteoporosis and Bone Mineral Salt Diseases Branch (16). Since then, numerous Chinese academic organizations have developed guidelines addressing prevention, diagnosis, treatment, fracture management, and traditional Chinese medicine (TCM) rehabilitation.

The quality and clinical applicability of these Chinese guidelines have not been systematically evaluated, making it difficult for clinicians to select appropriate ones and resulting in a gap between recommendations and real-world osteoporosis management. Awareness rates of osteoporosis among Chinese patients aged 40–49 and ≥50 years are only 0.9% and 7.0%, respectively (17). This study therefore reviewed and evaluated Chinese osteoporosis guidelines using the AGREE II and AGREE-REX tools to identify high-quality CPGs and assess the feasibility of their implementation in China.

## Materials and methods

### Design

We conducted this review following the 2020 Preferred Reporting Items for Systematic Review and Meta-Analysis statement. This review is part of an osteoporosis management guideline implementation project.

### Search strategy and guideline selection

The search strategy was developed in consultation with a statistics professor at ****** (W.Z.) to ensure that all relevant published CPGs were captured. Four electronic databases [China National Knowledge Infrastructure (CNKI), SinoMed, Wanfang Data, and VIP], a cross-database search platform (Medlive), and the website of the Chinese Medical Association were searched for guidelines published between 1 January 2014 and 31 December 2025. The reference lists of included guidelines were searched manually to identify any additional relevant guidelines. An example of a CNKI search is provided as **Supplementary Material 1**.

CPGs were included if they: (1) addressed osteoporosis prevention, treatment, or management in older adults; (2) met the Institute of Medicine’s definition of a guideline (18, 19); (3) were fully available in Chinese; and (4) were developed, updated, or revised after December 2013. Excluded records were: 1) guidelines for the diagnosis and treatment of osteoporosis comorbidities and 2) translations, simplified versions, historical versions, interpretations or explanations, reviews, and reports on appraisal methodology or guideline implementation.

After duplicate removal, two reviewers (S.S. and N.C.) independently performed initial eligibility screening based on the retrieved records’ titles and abstracts. Then, the full texts of potentially relevant CPGs were obtained and reviewed based on the inclusion and exclusion criteria. Any disagreement between the reviewers was resolved by a third reviewer (C.S.).

### Data extraction and appraisal

Two authors (S.S. and N.C.) independently collected the following information from each CPG: the title, purpose, institute of development, year of last available update, recipients, main recommendations, and evidence-based grading system used. Five reviewers (S.S., N.C., C.S., C.W., and L.G.) independently assessed the methodological and recommendation quality of all CPGs using the AGREE II and AGREE-REX, respectively.

As a globally recognized instrument, AGREE II assesses guideline development, reporting, and quality across 23 items grouped into six domains: scope and purpose, stakeholder involvement, rigor of development, clarity of presentation, applicability, and editorial independence (20). An additional item covers overall methodological quality (21). The scope and purpose domain encompasses the overall aim of the guidelines, health questions, and the target population. The stakeholder involvement domain is used to assess the extent to which the guidelines were developed by the appropriate stakeholders and represent the views of their intended users. The rigor of development domain relates to the processes used to gather and synthesize evidence and formulate and update recommendations. The clarity of presentation domain concerns the language, structure, and format of the guidelines. The applicability domain pertains to potential barriers to and facilitators of implementation, strategies used to improve uptake, and resource implications of guideline application. The editorial independence domain is used to identify the presence of undue bias from competing interests in recommendation formulation.

AGREE II items are scored on a 7-point scale ranging from 1 (strongly disagree) to 7 (strongly agree). Each of the five appraisers independently scored all items. Subsequently, a consensus meeting was held to resolve discrepancies. During the meeting, each item with discrepant scores was discussed in detail. Appraisers presented their rationale, and a consensus score was reached through deliberation. All appraisers had equal weight; no forced uniformity was imposed. If consensus could not be achieved after discussion, a third reviewer (C.S.) acted as arbitrator, and the final score was determined by majority vote. Minority opinions were documented but did not alter the final consensus score. This procedure was pre-defined and piloted during a calibration exercise. Independent scaled domain scores, expressed as percentages, were then obtained using the formula: Scaled domain score = (Obtained score − Minimum possible score)/(Maximum possible score − Minimum possible score) × 100%. Only CPGs with three or more AGREE II domain scores ≥ 50% were additionally assessed using the AGREE-REX.

The AGREE-REX evaluation was performed as a supplement to the AGREE II appraisal to assess recommendation credibility and implementability in clinical practice (22). As an adjunct to AGREE II, the AGREE-REX tool was used to gauge how credible and implementable the recommendations would be in real-world clinical settings. This instrument consists of nine items across three domains: clinical applicability, values and preferences, and implementability. This domain examines whether a CPG rests on a rigorous and complete appraisal of existing evidence, and to what extent its recommendations can be directly applied to orthopedic practice and osteoporotic patients. The values and preferences domain is used to assess whether target users’, patients’, policy/decision makers’, and guideline developers’ values and preferences were taken into consideration during guideline development to determine recommendation acceptability, feasibility for adoption in practice, and support by the health care system. The implementability domain concerns whether a CPG contains methodological suggestions for recommendation implementation in clinical practice and descriptions of the impacts of such implementation, which facilitate adoption.

AGREE-REX items are scored using a 7-point scale ranging from 1 (lowest quality) to 7 (highest quality). Items not applicable to osteoporosis management were given a score of 1 point. In accordance with the AGREE-REX instructions, items deemed not applicable to osteoporosis management were assigned a score of 1 point. We acknowledge that this scoring convention may lower domain scores for guidelines that did not address certain items. However, a score of 1 indicates the lowest quality on the scale and does not necessarily equate to poor overall guideline quality or distort the true quality assessment. This should be considered when interpreting the AGREE-REX results. The same consensus procedure was applied as AGREE II scoring. Domain scores were then calculated using the same formula as for AGREE II scores, and aggregated into a single quality score for each CPG.

### Statistical analysis

The statistical analysis was performed using SPSS (version 26.0; IBM Corporation, Armonk, NY, USA). Medians and interquartile ranges were calculated. Spearman’s correlations between domain scores were examined. *P* values < 0.05 were considered to be significant. To assess inter-rater reliability before the consensus process, we calculated intraclass correlation coefficients (ICCs) for each AGREE II and AGREE-REX domain using a two-way random-effects model for absolute agreement. The ICC values were interpreted as follows: <0.5, poor; 0.5–0.75, moderate; 0.75–0.9, good; and >0.90, excellent.

## Results

### CPG sample

The systematic search resulted in the retrieval of 576 publications (385 from electronic databases and 191 from relevant websites). The removal of duplicates and screening of titles and abstracts led to the exclusion of 498 publications; the remaining 78 publications underwent full-text review. Sixty-five publications were excluded for various reasons (**Figure 1**), and 13 CPGs were included in the analysis. These CPGs were produced by the Chinese Association of Gerontology and Geriatrics Osteoporosis Society (CAGG-OS) (6), Chinese Society of Osteoporosis and Bone Mineral Research (CSOBMR) (23, 24), CSOBMR Bone and Arthrology Group (25), Chinese Orthopaedic Association Group of Trauma Orthopedics (26), Chinese Orthopaedic Association (COA) (9), Chinese Society of General Practice (CSGP) (27), Chinese Rehabilitation Medicine Association (CRMA) (28, 29), Chinese Association of Integrated Traditional Chinese and Western Medicine Trauma Orthopedics Committee (CAITCWM-TOC) (30), Chinese Association of Chinese Medicine (CACM) (31), National Health Commission of China (32), G.X. Qiu et al. (33), and M.L. Qiu et al. (34).

**Figure 1:**
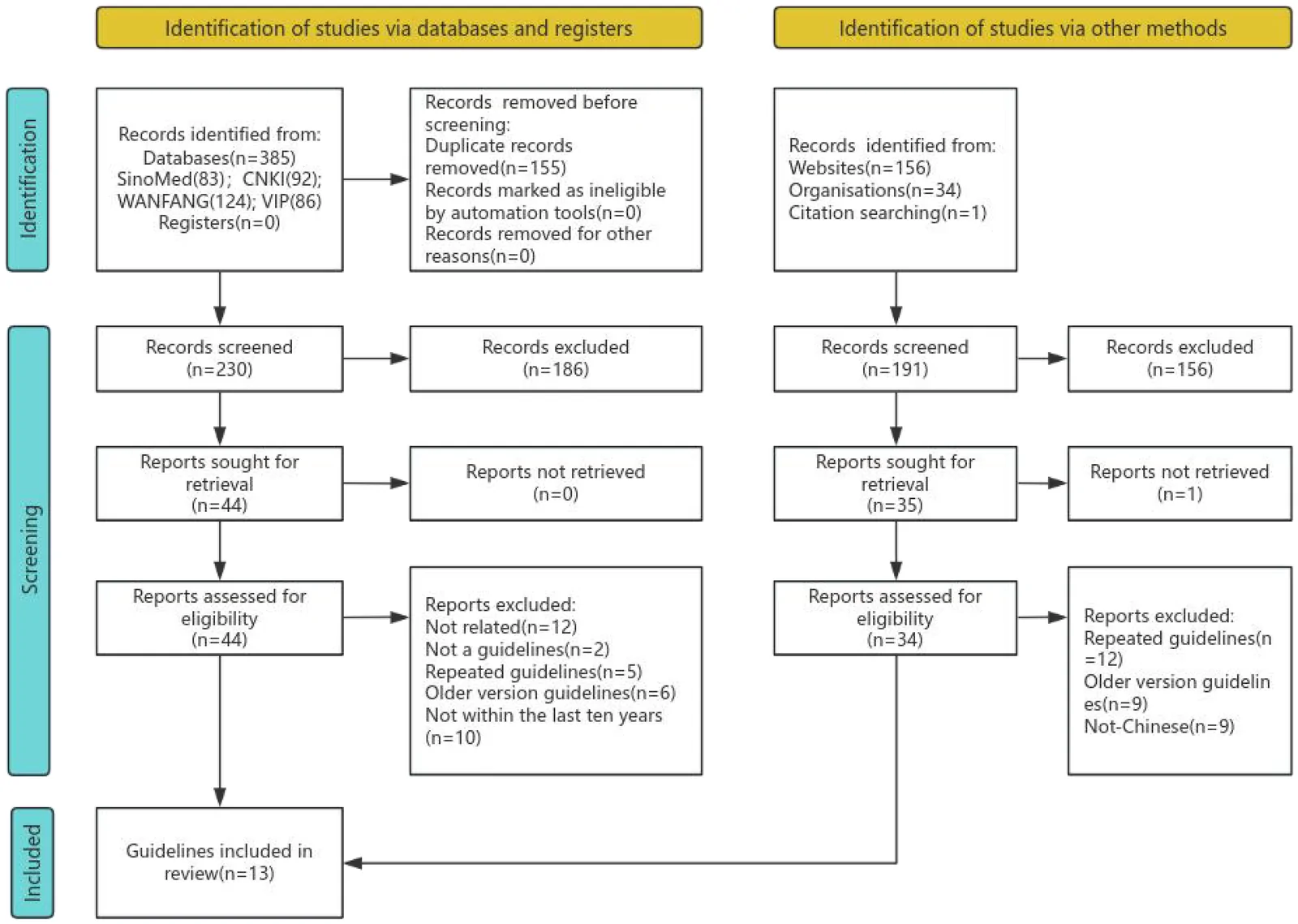
Diagram of guideline selection.

All but three of the CPGs were published in the last 5 years. The Grades of Recommendation, Assessment, Development, and Evaluation (GRADE) system was integrated into six of the CPGs. The other seven CPGs contain no reference to the use of any evidence-based grading system. Recommendations and other elements of the guidelines are shown in **Table 1** and **Supplementary Material 2**.

**Table 1:** Guideline characteristics.

Title	Institute/group	Year of last available update	Evidence-based grading system
China Guidelines for Diagnosis and Treatment of Senile Osteoporosis (2023) (6)	CAGG-OS	2023	GRADE
Guidelines for the Diagnosis and Treatment of Primary Osteoporosis(2022) (24)	CSOBMR	2022	No
Guidelines for Perioperative Intervention of Osteoporotic Fractures (25)	CSBOMR-BAG	2018	No
Guidelines for Standardized Anti-Osteoporosis Treatment after fragility fractures in China (26)	COA-TOG	2021	GRADE
Guidelines for the diagnosis and treatment of osteoporotic fractures (2022 edition) (9)	COA	2022	GRADE
Guideline for Diagnosis and Treatment of Osteoporosis in Men (23)	CSOBMR	2020	No
Guidelines for the primary care diagnosis and treatment of primary osteoporosis(2019) (27)	CSGP	2019	No
Guideline for Osteoporosis Rehabilitation (28, 29)	CRMA	2019	GRADE
Guidelines for Integrated Diagnosis and Treatment of Osteoporosis with Traditional Chinese and Western Medicine (30)	CAITCWM-TOC	2019	No
Guidelines for Traditional Chinese Medicine Diagnosis and Treatment of Osteoporosis Fractures (31)	CACM	2023	GRADE
Guidelines for the Diagnosis, Treatment and Management of Elderly Hip Fractures (2022 edition) (32)	NHCC	2022	No
Guide to Diagnosis and Treatment of Osteoporotic Fracture in China—Osteoporotic Fracture Diagnosis and Treatment Principles (33)	G.X. Qiu et al.	2018	No
Practice guideline for patients with osteoporosis (34)	M.L. Qiu et al.	2020	GRADE

CAGG-OS, Osteoporosis Society of China’s Association of Gerontology and Geriatrics; GRADE, Grades of Recommendation, Assessment, Development, and Evaluation; CSOBMR-BAG, Bone and Arthrology Group of the Chinese Society of Osteoporosis and Bone Mineral Research; COA-TOG, Orthopaedic Trauma Group of the Chinese Orthopaedic Association; CSGP, Chinese Society of General Practice; CRMA, China Rehabilitation Medicine Association; CAITCWM-TOC, Chinese Association of Integrated Traditional Chinese and Western Medicine Trauma Orthopedics Committee; CACM, Chinese Association of Chinese Medicine; NHCC, National Health Commission of China.

### AGREE II results

Across all CPGs, the median overall quality score was 54% (interquartile range: 49%–71%), with four guidelines achieving >50% in every domain (**Figure 2**). The CRMA guidelines achieved the highest score (95%) and the guidelines of G.X. Qiu et al. received the lowest score (35%). Among domains, the median rigor of development score was lowest[38% (25%, 94%)] and the median stakeholder involvement and clarity of presentation score was highest [72% (44%, 72%); 72% (61%, 83%)]. Most CPGs that employed the GRADE system (CAGG-OS, COA-GTO, COA, CRMA, and M.L. Qiu et al.) scored >79% in the rigor of development domain.

**Figure 2:**
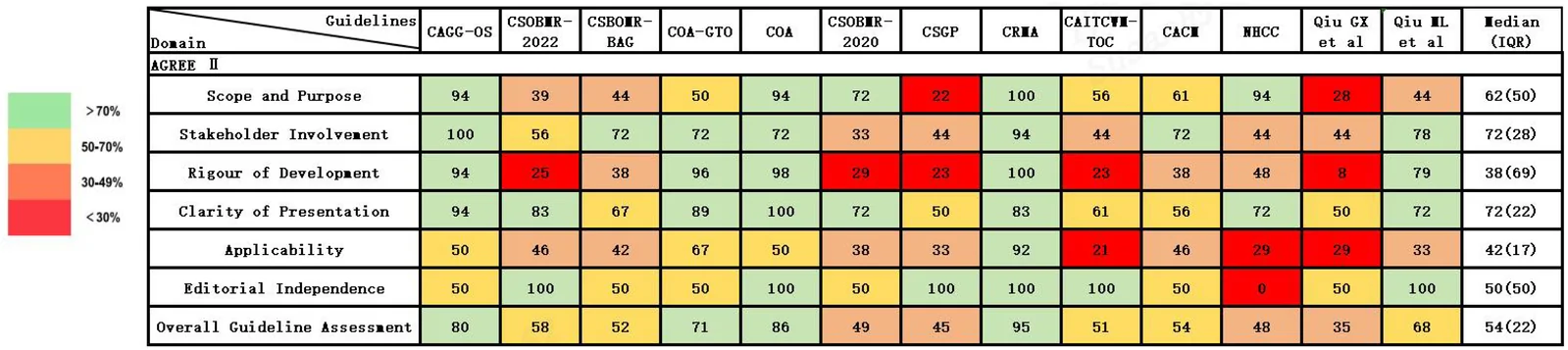
AGREE II scores. Each cell shows the scaled score (%) of one guideline (column) in one AGREE II domain (row); the cell color indicates the quality level (green, >70%; yellow, 50–70%; orange, 30–49%; red, <30%). The last column presents the median (interquartile range, IQR) of each domain across all guidelines. AGREE II, Appraisal of Guidelines for Research and Evaluation II. Guideline abbreviations are defined in **Table 1**.

Scores were highest (>70%) for the following items: “The guideline development group includes individuals from all relevant professional groups” (stakeholder involvement), “The population (patients, public, etc.) to whom the guideline is meant to apply is specifically described” (scope and purpose), “There is an explicit link between the recommendations and the supporting evidence” (rigor of development), “easy to identify key recommendations” (clarity of presentation), and “The views of the funding body have not influenced the content of the guideline” (editorial independence). Nine of the 13 guidelines (69.2%) did not mention the application of monitoring or auditing criteria, and seven (53.8%) did not mention external expert review. In terms of mean scaled item scores, these items scored 9.2% and 29.7%, respectively. In addition, the interests of the guideline development group members were not disclosed in five CPGs (CSBOMR-BAG, CSOBMR-2020, CACM, NHCC, and G.X. Qiu et al.). Some guidelines (CSOBMR-2023, CSOBMR-2020, CSGP, and G. X. Qiu et al.) did not describe the literature search strategy used. The CAITCWM-TOC and CACM guidelines mentioned that evidence was retrieved but did not specify the search strategy. Seven guidelines (CSOBMR-2023, CSOBMR-2020, CSGP, CAITCWM-TOC, CACM, NHCC, and G.X. Qiu et al.) explicitly acknowledged the lack of a procedure for updating.

### AGREE-REX results

Ten CPGs with three or more AGREE II domain scores ≥ 50% were assessed using the AGREE-REX. The median overall recommendation quality score was 56% (32%, 63%), and four CPGs scored <30% in one or more domains (**Figure 3**). The overall quality score was lowest for the CSOBMR-2022 guidelines (26%). Among AGREE-REX domains, the median values and preferences score was lowest [43% (18%, 52%)]. The CPG of the CRMA, which focuses on functional assessment, rehabilitation, environmental modification, and risk prevention, achieved the highest overall (87%) and domain scores.

**Figure 3:**
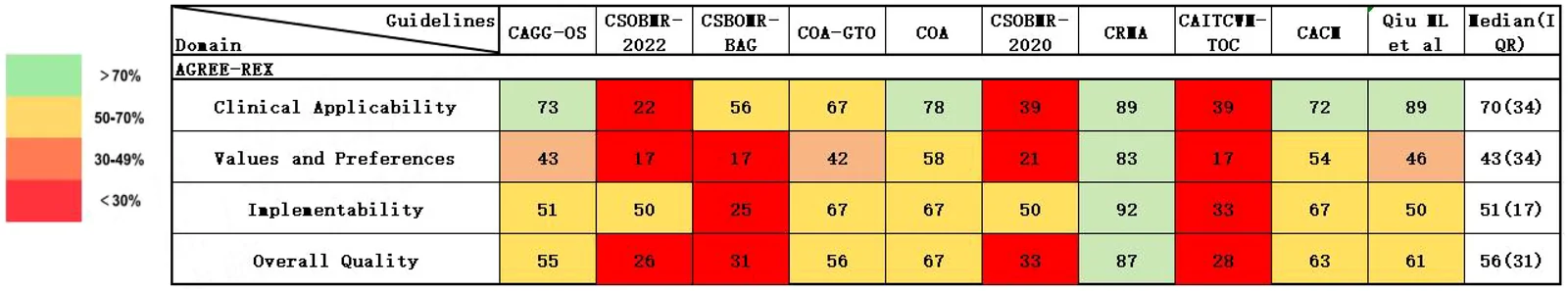
AGREE-REX scores. Each cell shows the scaled score (%) of one guideline (column) in one AGREE-REX domain (row); the cell color indicates the quality level (green, >70%; yellow, 50–70%; orange, 30–49%; red, <30%). The last column presents the median (interquartile range, IQR) of each domain across the assessed guidelines. AGREE-REX, Appraisal of Guidelines for Research and Evaluation—Recommendation Excellence. Guideline abbreviations are defined in **Table 1**.

The median score for the “applicability to target users” item (clinical applicability domain) was 83% (42%, 99%), and that for the “purpose” item (implementability domain) was 83% (33%, 85%). Scores for items in the values and preferences domain were consistently lower, ranging from 33% to 44%, with a median of 42%. Seven (70%) of the guidelines evaluated described the consideration of recommendation strength, harm-benefit trade-offs, and anticipated impacts on patient outcomes such as bone density, fracture risk, and quality of life. However, most [8 of 10, 80%] guidelines did not document whether the values and preferences of policy/decision makers or guideline developers were considered during recommendation formulation.

### Correlations between AGREE-REX and AGREE II scores

A positive correlation emerged between the overall quality scores of AGREE II and AGREE-REX (r = 0.665). Notably, the AGREE-REX overall score was strongly linked to the AGREE II rigor of development domain (r = 0.839). The AGREE-REX clinical applicability domain score correlated positively with the AGREE II rigor of development (*r* = 0.826) and overall quality (*r* = 0.744) scores. No significant negative correlation between scores was observed (**Figure 4**).

**Figure 4:**
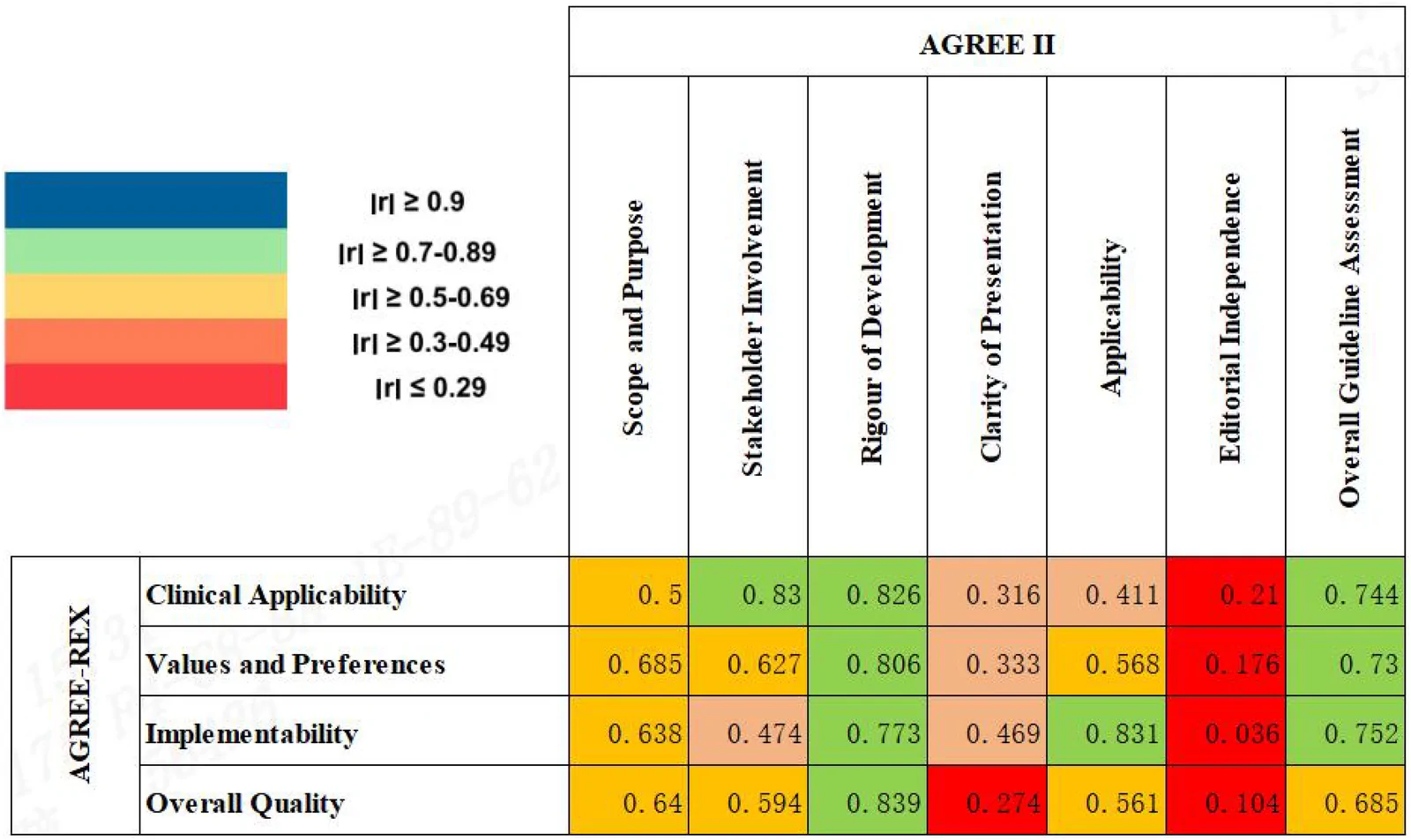
Correlations between AGREE II and AGREE-REX score. The cell color indicates the absolute strength of the correlation (dark blue, |r| ≥ 0.9; green, |r| = 0.7–0.89; yellow, |r| = 0.5–0.69; orange, |r| = 0.3–0.49; red, |r| ≤ 0.29).

## Discussion

As part of a broader implementation project for osteoporosis management guidelines, this study provides an overview of the methodological and recommendation quality of Chinese CPGs in this field, as well as their implementation feasibility. Although the quality of the included CPGs varies, the finding suggest that implementation is feasible. This appraisal support informed and transparent decision-making in guideline selection and adaptation.

According to the AGREE II assessment, the methodological quality of Chinese osteoporosis CPGs is moderate. High-scoring items indicate that Chinese developers prioritize transparency in the development process, clear identification of target users, and the reliability and clarity of recommendations. However, scores in the domains of editorial independence, applicability, and rigor of development were relatively low. Guidelines that did not adopt the GRADE system generally performed worse in these domains and were excluded from AGREE-REX evaluation. Notably, guidelines developed with input from evidence-based medicine centers, such as those from the CRMA, were more prescriptive. Based on these results, we suggest that:1) CPG developers should consider potential consequences at both organizational and health system levels; 2) in addition to GRADE, alternative evidence grading systems such as those from SIGN (35) or the University of Iowa (36) could be adopted; and 3) involving evidence-based medicine centers in guideline development may improve methodological quality. Our results strongly support the adoption of GRADE or equivalent evidence-grading systems to improve the reliability of future Chinese osteoporosis guidelines.

Our AGREE-REX evaluation of ten CPGs revealed that the overall quality of osteoporosis management recommendations in Chinese guidelines requires improvement. The distribution of high AGREE-REX item scores indicates that the developers of Chinese osteoporosis guidelines attach great importance to the applicability of recommendations to target users’ environments and the achievability of implementation goals. Most recommendations address issues related to osteoporosis management about which target users are concerned, such as the evaluation of various physical functions, calcium supplementation, fall prevention, and exercise and nutritional interventions, as well as the impacts of recommendation adoption on patient factors. However, the consideration of anticipated impacts on organizations and systems is insufficient, as reflected by the low values and preferences domain and item scores. The values and preferences of policy stakeholders impact the implementation of guideline recommendations in health care systems (36). Due to its high incidence and close correlation with populational aging, osteoporosis is a social issue that requires a high level of governmental attention (3, 37). The full consideration of policy/decision makers’ values and preferences during guideline development can help to promote the investment of public resources in osteoporosis management. Based on the AGREE-REX assessment results, we offer the following suggestions. Recommendation formulation should account for potential consequences on organizations and healthcare systems as a whole. Moreover, beyond addressing the values of end-users and patients, CPG developers need to incorporate the perspectives of policy-makers and decision-makers-–this would help secure greater public awareness, funding, and resources for osteoporosis care.

The development of Chinese osteoporosis guidelines involves multiple stakeholders and comprehensive content. Because osteoporosis is systemic and presents various symptoms, its management requires input from multiple specialties, including orthopedics, geriatrics, endocrinology, rehabilitation, and TCM (38). This study highlights the active involvement of diverse professional groups, which enhances guideline comprehensiveness. Government bodies such as the National Health Commission have also participated in guideline development, reflecting national recognition of osteoporosis as a priority. The ten guidelines evaluated with AGREE-REX focus on prevention, nutrition, exercise, psychological support, rehabilitation, and risk reduction, aligning with international guidelines from SIGN (39), NICE (40), NOGG (41), and Osteoporosis Canada (42). This alignment underscores the relevance of Chinese guidelines to global osteoporosis management efforts. Chinese guidelines are evidence-based and tailored to the local population, recommending functional assessments, diet, exercise, rehabilitation, and environmental modifications. Some CPGs include TCM practices such as tai chi and qigong (e.g., Eight Section Brocade, Five Animal Play), and discuss anticipated benefits like pain relief, improved muscle strength, and better gait. However, due to limited clinical evidence, the strength of these recommendations is often rated as “C.” Our analysis indicates that: 1) Chinese guideline developers have effectively integrated their professional expertise; 2) recommendations consider local demographic, disease, and environmental characteristics; and 3) TCM-based recommendations are culturally appropriate but require more evidence to confirm their reliability.

Several limitations should be acknowledged. First, restricting the search to CPGs published from 2014 onward may have excluded earlier but still relevant guidelines. Second, using a cutoff of three or more AGREE II domains scoring ≥50% for AGREE-REX assessment may have omitted some methodologically sound CPGs. This limitation should be considered when interpreting our findings, and future reviews might adopt alternative inclusion strategies to capture a broader range of guidelines. Third, although the rigor of development domain is important, it was not used as a separate inclusion criterion. Finally, our inclusion criteria excluded guidelines focused on osteoporosis comorbidities. These excluded guidelines may still contain valuable recommendations for specific dimensions of osteoporosis care, and our evaluation results should not be interpreted as representing the quality of all Chinese osteoporosis guidelines. Future reviews with broader inclusion criteria are warranted. We also acknowledge that new epidemiological data published between 2024 and 2025 were not systematically included, as the primary aim of this review was to appraise guideline quality rather than to update epidemiological estimates. Future updates of this review could incorporate the most recent epidemiological evidence to contextualize guideline development.

Finally, this study evaluated the methodological and recommendation quality of guidelines but did not assess the association between guideline quality and clinical outcomes. Whether higher-quality guidelines lead to better clinical outcomes remains unclear. High-quality guidelines may be more implementable and may improve care processes, but direct evidence linking AGREE II or AGREE-REX scores to patient outcomes is lacking. Future studies should prospectively evaluate the impact of guideline implementation on clinical endpoints such as fracture rates, quality of life, and mortality.

## Conclusion

Our findings indicate that both the methodological and recommendation quality of Chinese osteoporosis CPGs are only moderate, highlighting a clear need for further enhancement. We recommend the adoption of guideline formulation systems to improve guideline rigor. More consideration of the values and preferences of various stakeholders, especially guideline developers and policy/decision makers, during recommendation formulation is also needed. In particular, actively involving policy-makers and decision-makers in the development process is essential to ensure that recommendations are feasible within the broader healthcare system and to secure necessary public resources. Although the quality of the CPGs examined in this study varies, the recommendations for functional assessment, diet and exercise, rehabilitation, and environmental modification contribute to the management of osteoporosis. The recommendations for traditional Chinese medicine exercises in these guidelines could eventually serve as a reference for CPG recommendation formulation in other countries, but more evidence for their outcomes is needed to improve reliability.

## Data Availability

The original contributions presented in the study are included in the article/**Supplementary Material**. Further inquiries can be directed to the corresponding author.
