# Supplementary material for: Quality of osteoporosis management guidelines produced in China: a systematic review
Source: Front Endocrinol (Lausanne). 2026 Oct 15;17:1907308. doi: 10.3389/fendo.2026.1907308 (PMC13716612; doi:10.3389/fendo.2026.1907308)
Supplement: Supplementary file 1 [file Table1.docx]

1.Database: CNKI(Chinese)

2.Database URL: https://www.cnki.net/

3.Search term: osteoporosis，guideline；

4.Search strategy：(Title: Osteoporosis (Precision)) AND (Title: Guidelines (Precision)) NOT (Title: Interpretation (Precision))
