# Supplementary material for: Quality of osteoporosis management guidelines produced in China: a systematic review
Source: Front Endocrinol (Lausanne). 2026 Oct 15;17:1907308. doi: 10.3389/fendo.2026.1907308 (PMC13716612; doi:10.3389/fendo.2026.1907308)
Supplement: Supplementary file 2 [file Table2.docx]

| ON. | Guideline name | Clinical problem | Recommendation (related to disease management) | Evidence level | Evaluation domains | Evaluation items | Expert consensus evaluation basis | | | Quality score |
| --- | --- | --- | --- | --- | --- | --- | --- | --- | --- | --- |
|  |  |  |  |  |  |  | qualified items | unqualified items | Unrelated items |  |
| 1 | CAGG-OS | How to supplement calcium supplements for elderly patients with osteoporosis? | Elderly patients with osteoporosis generally suffer from insufficient or deficient calcium and vitamin D. Combining calcium and vitamin D supplementation can increase bone density in the lumbar spine and femoral neck, slightly reducing the risk of hip fractures. The total daily intake of elemental calcium for elderly osteoporosis is 1000~1200 mg, with a tolerable maximum of 2000 mg. In addition to dietary supplementation, an additional 500~600 mg of elemental calcium is needed daily. The selection of calcium supplements should consider the content, safety, effectiveness, and compliance of elemental calcium | 1B | Clinical Applicability | Evidence | 1. Consistency of results: The Chinese Dietary Guidelines for Residents (2022) recommend a daily calcium intake of 1000 mg for elderly people (>65 years old). The Diagnosis and Treatment Guidelines for Primary Osteoporosis (2022) recommend that middle-aged and elderly people aged 50 and above should have a daily intake of 1000-1200mg of elemental calcium.  2. Directional evidence of clinical or health issues: reported outcome indicators such as bone density, fracture risk, BTM, bone resorption, and bone turnover;  3. Advantages and disadvantages: Reported the advantages and disadvantages of calcium supplements and exposure to sunlight. | 1.1: The Chinese Dietary Guidelines for Residents (2022) recommend a daily calcium intake of 1000 mg for elderly people (>65 years old). The Diagnosis and Treatment Guidelines for Primary Osteoporosis (2022) recommend that middle-aged and elderly people aged 50 and above should have a daily intake of 1000-1200mg of elemental calcium.  1.3: Reported outcome indicators such as "bone density", "fracture risk", BTM, bone resorption index, and bone transformation index;  1.5: Reported the pros and cons of calcium supplements and exposure to sunlight. | 2.7 | 3 |
|  |  |  | Patients with hypercalcemia or hypercalcemia should not use calcium supplements | 1C |  | Applicability to Target Users | 2.1: The issue of supplementing calcium supplements for osteoporosis patients;  2.2; Patients with osteoporosis;  2.3: Supplemental dosage of calcium supplements and methods to promote calcium absorption;  2.5: The certainty or strength of recommended opinions should be consistent with the trade-off between advantages and disadvantages. | 2.4: The trade-off between the use of calcium supplements in patients with "hypercalcemia" and "hypercalcemia" was not mentioned. |  | 6 |
|  |  |  | It is recommended to expose oneself to more sunlight to promote the formation of endogenous vitamin D in the skin, thereby promoting calcium absorption in the intestines | 1B |  | Applicability to Patients/Populations | 3.1: "Bone density", "fracture risk", BTM, bone resorption index, and bone transformation index;  3.2: Increase bone density, reduce the risk of hip fractures and any type of fractures, slightly decrease BTM, and inhibit the expression of bone transition marker NTX;  3.4: Patients with hypercalcemia or hypercalcemia should not use calcium supplements. | 3.3:Recommendation for determining the importance of unreported patient outcomes. |  | 6 |
|  |  |  |  |  | Values and Preferences | Values and Preferences of Target Users | 4.1: Only adverse reactions were mentioned | 4.2: Not mentioned;  4.3: Some recommended opinions have a certain degree of clinical flexibility and are suitable for different individual patients, making it easier for clinical decision-making; Some recommendations are not very suitable for clinical decision-making: although the recommendations specify the population that prohibits calcium supplements, the role of recommendations in clinical decision-making has not been reported;  4.4: Not mentioned. |  |  |
|  |  |  |  |  |  | Values and Preferences of Patients/Populations | 5.2: The selection of calcium agents needs to consider safety, efficacy, and compliance. | 5.1: Not mentioned;  5.3: Not distinguished;  5.4: Not mentioned. |  | 3 |
|  |  |  |  |  |  | Values and Preferences of Policy/Decision-Makers |  | 6.3: Not mentioned. | 6.1  6.2  6.4 | 1 |
|  |  |  |  |  |  | Values and Preferences of Guideline Developers |  | 7.1: Not mentioned;  7.2: Not mentioned;  7.3: Not mentioned. |  | 1 |
|  |  |  |  |  | Implementability | Purpose | 8.1: Calcium supplementation is consistent with the implementation goals (diagnosis and treatment of elderly osteoporosis);  8.2: Mention the expected effects of adopting recommended opinions on patient bone density, hip and any part of the fracture, reduction of BTM, and inhibition of bone transition marker NTX expression. |  |  | 6 |
|  |  |  |  |  |  | Local Application and Adoption | 9.1: Explained the different situations where "the calcium content in the daily diet of the Chinese population is usually low", as well as the different effects of dairy products, calcium, and vitamin D supplementation on "postmenopausal women" and "elderly people in the community". | 9.2: Not mentioned  9.3: Not mentioned;  9.4: Not mentioned. |  | 2 |
|  |  | How can elderly patients with osteoporosis prevent falls? | It is recommended to conduct a fall risk assessment for elderly patients with osteoporosis and fragile fractures. Interventions that improve balance and/or include a comprehensive exercise program should be provided for patients at risk. | 1B | Clinical Applicability | Evidence | 1.2: The research results of SIGN and NOGG are consistent;  1.3: The precise intervention measures for preventing falls were mentioned, with the population being the elderly and the outcome being falls;  1.5: Refers to the pros and cons, such as side injuries caused by excessive or inappropriate exercise. | 1.1: Not mentioned;  1.4: Not mentioned;  1.6: Not mentioned;  1.8: Not mentioned. | 1.7 | 3 |
|  |  |  | The measures for preventing falls in elderly patients with osteoporosis include regular exercise, selecting appropriate clothing and shoes, scientifically selecting and using age appropriate assistive devices, carrying out aging friendly home environment renovation, regularly conducting fall prevention assessments, and following medical advice for medication. | 2B |  | Applicability to Target Users | 2.1: Recommended preventive measures for falls address hygiene issues such as "How to prevent falls in elderly patients with osteoporosis";  2.2: The scope of user practice for osteoporosis prevention and medication orders is consistent with the target patient;  2.3: The recommended opinions are consistent with the user's health education content for the target patient;  2.4: Consistent recommendations and trade-offs. | 2.2 and 2.3, there may be a possibility that the recommended opinions exceed the scope of user practice;  2.5: Not mentioned. |  | 5 |
|  |  |  |  |  |  | Applicability to Patients/Populations | 3.1: Relevant outcomes have been considered, such as reducing the incidence of fractures, disability, and mortality rates;  3.2: Implementation of recommendations can affect leg improvement, muscle strength, improving walking stability, etc;  3.4: The recommendation mentions the precautions for patients applying drugs that act on the central nervous system, cardiovascular system, etc. | 3.3: The determination methods for muscle strength, walking stability, and the risk of falls were not mentioned. |  | 5 |
|  |  |  |  |  | Values and Preferences | Values and Preferences of Target Users |  | 4.1: Not mentioned;  4.2: Not mentioned;  4.3: Not mentioned;  4.4: Not mentioned. |  | 1 |
|  |  |  |  |  |  | Values and Preferences of Patients/Populations | 4.1: Conducted a certain degree of discussion on the patient's values and preferences;  4.3: There are related claims, such as: strength training can improve the strength of leg muscles, thereby enhancing stability during walking. | 4.2: Not mentioned;  4.3: Not mentioned. |  | 3 |
|  |  |  |  |  |  | Values and Preferences of Policy/Decision-Makers |  |  | 6.1，6.2，6.3，6.4 | 1 |
|  |  |  |  |  |  | Values and Preferences of Guideline Developers |  | 7.1: Not mentioned;  7.2: Not mentioned;  7.3: Not mentioned. |  | 1 |
|  |  |  |  |  | Implementability | Purpose | 8.1: The recommended opinion is about fall prevention in patients with osteoporosis, consistent with the goals of the guidelines;  8.2: Described the impact of adopting recommendations on patients, such as improving leg muscle strength and improving walking stability. | 8.2: The expected impact of adopting recommendations on target users, organizations, or systems is not mentioned. |  | 6 |
|  |  |  |  |  |  | Local Application and Adoption |  | 9.1: Not mentioned;  9.2: Not mentioned;  9.3: Not mentioned;  9.4: Not mentioned. |  | 1 |
|  |  | How to provide exercise intervention for elderly patients with osteoporosis? | It is recommended that elderly patients with osteoporosis follow the principles of individualization (exercise mode, frequency, time, and intensity), capacity based, and gradual progress, and regularly engage in some moderate to low-intensity diversified exercises (aerobic exercise, muscle strengthening, balance training, etc.) to maintain moderate improvement of existing functions. | 1B | Clinical Applicability | Evidence | 1.2: It is mentioned that multiple systematic evaluations have confirmed the positive effects of exercise intensity, exercise mode, and exercise type on the bones of postmenopausal women, and the results are consistent;  1.3: Considered the types, amounts, and benefits of exercise related to health issues;  1.5: The impact of progressive resistance training on the risk of falls and the bone density of the lumbar and total hip joints is not yet clear. | 1.1: Not mentioned;  1.4: Not mentioned;  1.6: Not mentioned;  1.7: Not mentioned;  1.8: Not mentioned. |  | 3 |
|  |  |  | If physical conditions permit, regularly engage in weight-bearing exercises to enhance muscle strength and prevent falls. | 2C |  | Applicability to Target Users | 2.1: The recommended exercise plan addresses hygiene issues, such as how to intervene in exercise for elderly patients with osteoporosis;  2.2: The user practice scope of health guidance for exercise intervention in osteoporosis is consistent with the target patients;  2.3: The recommended opinions are consistent with the user's health education content for the target patient;  2.4: Consistent recommendations and trade-offs;  2.5: Based on the level of risk associated with exercise, the recommended intensity has been determined. | 2.2 and 2.3, there may be a possibility that the recommended opinions exceed the scope of user practice. |  | 6 |
|  |  |  | It is recommended that elderly patients with osteoporosis engage in at least 150-300 minutes of moderate intensity exercise per week, or 75-150 minutes of high-intensity aerobic exercise per week. | 2C |  | Applicability to Patients/Populations | 3.1: Relevant outcomes have been considered, such as bone mass, bone density, and falls;  3.2: Recommended opinions can affect the improvement of limb function, enhance muscle strength, and prevent falls;  3.4: Recommendation mentions different exercise plans for combined lower limb osteoarthritis and chronic diseases. | 3.3: The methods for determining bone density, balance, and muscle strength were not mentioned. |  | 5 |
|  |  |  | Elderly patients with osteoporosis often have lower limb osteoarthritis, and it is not recommended to engage in activities such as squatting, climbing stairs, or mountain climbing to avoid side injuries caused by excessive or inappropriate movements such as bending or twisting. | 2C | Values and Preferences | Values and Preferences of Target Users | 4.3: Recommendation: Personalized plans were recommended for complications. | 4.1: Not mentioned;  4.2: Not mentioned;  4.4: Not mentioned. |  | 2 |
|  |  |  |  |  |  | Values and Preferences of Patients/Populations | 5.1: Considering the patient's quality of life and adverse reactions;  5.4: Fully describe the benefits of exercise. | 5.2: Not mentioned;  5.3: Not mentioned. |  | 4 |
|  |  |  |  |  |  | Values and Preferences of Policy/Decision-Makers |  |  | 6.1，6.2，6.3，6.4 | 1 |
|  |  |  |  |  |  | Values and Preferences of Guideline Developers |  | 7.1: Not mentioned;  7.2: Not mentioned;  7.3: Not mentioned. |  | 1 |
|  |  |  |  |  | Implementability | Purpose | 8.1: The recommended opinion is about exercise intervention for osteoporosis, which is consistent with the goals of the guidelines;  8.2: Describing the impact of adopting recommendations on patients, such as improving limb function, enhancing muscle strength, and preventing falls. | 8.2: The expected impact of adopting recommendations on target users, organizations, or systems is not mentioned. |  | 6 |
|  |  |  |  |  |  | Local Application and Adoption |  | 9.1: Not mentioned;  9.2: Not mentioned;  9.3: Not mentioned;  9.4: Not mentioned. |  | 1 |
|  |  | How to provide nutritional intervention for elderly patients with osteoporosis? | It is recommended that elderly patients with osteoporosis consume a balanced diet containing high-quality protein, rich minerals, and vitamins, and consume sufficient amounts of protein. | 1B | Clinical Applicability | Evidence | 1.2: The recommendation mentions the expert consensus on nutrition and exercise management for patients with primary osteoporosis and the similar dosage of protein supplementation in the Chinese Dietary Guidelines for Residents (2022);  1.3: Recommendation mentions the supplementation of protein and calcium containing foods, the population involved, and related benefits and outcomes, such as reducing the risk of fractures and increasing bone density;  1.8: The recommended opinion mentions the dose-response gradient of the duration of smoking and the amount of alcohol consumed. | 1.1: Not mentioned;  1.4: Not mentioned;  1.5: Not mentioned;  1.6: Not mentioned. | 1.7 | 3 |
|  |  |  | Recommend elderly patients with osteoporosis to consume more calcium rich foods, such as dairy and its products, soy and its products, aquatic products, nuts, dark green vegetables, etc. | 1B |  | Applicability to Target Users | 2.1: The recommended opinions have addressed the hygiene issues of target users regarding how to provide nutritional intervention for elderly osteoporosis patients;  2.2: The plan for nutritional intervention in the recommended opinions is consistent with the practical scope of the target users;  2.3: A high protein diet and a high calcium diet are associated with reducing the risk of fractures; Smoking and drinking are associated with an increased risk of fractures;  2.4: The certainty and trade-off of a high protein diet, high calcium diet, smoking cessation and alcohol restriction are consistent. |  |  | 7 |
|  |  |  | Suggest elderly patients with osteoporosis to quit smoking and limit alcohol consumption | 2C |  | Applicability to Patients/Populations | 3.1: The guidelines include outcomes related to the target patient, such as fracture risk and bone density;  3.2: Mention the impact of high protein and high calcium diets, smoking cessation and alcohol restriction on related outcomes. | 3.3: Not mentioned;  3.4: Not mentioned. |  | 4 |
|  |  |  |  |  | Values and Preferences | Values and Preferences of Target Users |  | 4.1: Not mentioned;  4.2: Not mentioned;  4.3: Not mentioned;  4.4: Not mentioned. |  | 1 |
|  |  |  |  |  |  | Values and Preferences of Patients/Populations | 5.1: Conducted a certain degree of discussion;  5.4: Describing the benefits of decision-making in reducing fracture risk and increasing bone density. | 5.2: Not mentioned;  5.3: Not mentioned. |  | 3 |
|  |  |  |  |  |  | Values and Preferences of Policy/Decision-Makers |  |  | 6.1，6.2，6.3，6.4 | 1 |
|  |  |  |  |  |  | Values and Preferences of Guideline Developers |  | 7.1: Not mentioned;  7.2: Not mentioned;  7.3: Not mentioned. |  | 1 |
|  |  |  |  |  | Implementability | Purpose | 8.1: The recommended opinion is about the nutrition of osteoporosis, consistent with the goals of the guidelines;  8.2: Described the impact of adopting recommendations on patients, such as reducing fracture risk and improving bone density. | 8.2: The expected impact of adopting recommendations on target users, organizations, or systems is not mentioned. |  | 6 |
|  |  |  |  |  |  | Local Application and Adoption | 9.1: In Eastern countries, a healthy dietary pattern can significantly reduce the risk of fractures. | 9.2: Not mentioned;  9.3: Not mentioned;  9.4: Not mentioned. |  | 2 |
|  |  | How to provide health education and psychological intervention for elderly patients with osteoporosis? | It is recommended to conduct systematic and regular health education for elderly patients with osteoporosis and brittle fractures, increase their understanding of the disease, including informing them of the risk factors, hazards, and prevention of osteoporosis, the importance of improving lifestyle, medication knowledge and monitoring, etc., to improve treatment compliance. | 1B | Clinical Applicability | Evidence | 1.2: Multiple research results have consistency;  1.3: The guidelines consider the beneficial outcomes of clinical issues, such as negative attitudes such as fear, anxiety, depression, physical activity and function, and quality of life. | 1.1: Not mentioned;  1.3: Incomplete description of exact intervention measures;  1.4: Not mentioned;  1.5: Not mentioned;  1.6: Not mentioned. | 1.7，1.8. | 2 |
|  |  |  | It is recommended to provide continuous psychological communication and support for elderly patients with osteoporosis and fragile fractures, to help alleviate anxiety, overcome fear of falls and fractures, and improve treatment compliance and quality of life. Recommend administering antidepressant and anxiety medication according to the situation. | 2C |  | Applicability to Target Users | 2.1: The recommended opinions have solved the hygiene problem of the target user "how to provide health education and psychological intervention for elderly osteoporosis patients?";  2.2: Health education and psychological intervention in the recommended opinions are consistent with the practical scope of the target users;  2.3: Action on practical scope and recommended opinions, such as; Health education courses, psychological counseling, etc. have consistency.  2.4: The direction of recommendations should be consistent with the pros and cons;  2.5: The recommended strength should be balanced against the pros and cons. |  |  | 7 |
|  |  |  |  |  |  | Applicability to Patients/Populations | 3.1: Guidelines include outcomes related to the target patient, such as negative attitudes such as fear, anxiety, depression, physical activity and function, and quality of life;  3.2: Mention the impact of health education, psychological communication, and psychological support on related outcomes. | 3.3: Not mentioned;  3.4: Not mentioned. |  | 4 |
|  |  |  |  |  | Values and Preferences | Values and Preferences of Target Users |  | 4.1: Not mentioned;  4.2: Not mentioned;  4.3: Not mentioned;  4.4: Not mentioned. |  | 1 |
|  |  |  |  |  |  | Values and Preferences of Patients/Populations | 5.1: Conducted a certain degree of discussion;  5.4: Describing the benefits of decision-making in improving patients' negative attitudes, reducing fear of falls, and improving quality of life. | 5.2: Not mentioned;  5.3: Not mentioned. |  | 3 |
|  |  |  |  |  |  | Values and Preferences of Policy/Decision-Makers |  |  | 6.1，6.2，6.3，6.4 | 1 |
|  |  |  |  |  |  | Values and Preferences of Guideline Developers |  | 7.1: Not mentioned;  7.2: Not mentioned;  7.3: Not mentioned. |  | 1 |
|  |  |  |  |  | Implementability | Purpose | 8.1: The recommended opinion is about psychological intervention and health education for osteoporosis, consistent with the goals of the guidelines;  8.2: Describing the impact of adopting recommendations on patients, such as improving their negative mindset, reducing fear of falls, and improving their quality of life. | 8.2: The expected impact of adopting recommendations on target users, organizations, or systems is not mentioned. |  | 6 |
|  |  |  |  |  |  | Local Application and Adoption |  | 9.1: Not mentioned;  9.2: Not mentioned;  9.3: Not mentioned;  9.4: Not mentioned. |  | 1 |
|  |  | How can elderly patients with osteoporosis undergo traditional Chinese medicine and rehabilitation treatment? | Elderly patients with osteoporosis and brittle fractures should undergo active rehabilitation treatment. Common rehabilitation measures include lifestyle interventions (diet, outdoor activities, improving bad habits, etc.) and medical interventions (rehabilitation training and physical therapy). | 2C | Clinical Applicability | Evidence | 1.2: Refers to multiple studies with consistent results;  1.3: The recommendation takes into account the precise measures and benefits of traditional Chinese medicine rehabilitation treatment, such as low-intensity exercise, electrotherapy, magnetic therapy, etc. The outcomes include enhancing muscle strength and preventing falls. | 1.1: Not mentioned;  1.4: Not mentioned;  1.5: Not mentioned;  1.6: Not mentioned;  1.8: Not mentioned. | 1.7 | 2 |
|  |  |  | Elderly patients with osteoporosis can choose exercise forms and intensity that are suitable for their own conditions for rehabilitation training. It is recommended to use traditional exercise methods such as Tai Chi, Baduan Brocade, and Wuqinxi to relieve pain, enhance muscle strength, improve gait, and enhance limb function. | 2C |  | Applicability to Target Users | 2.1: The recommended opinions have solved the hygiene problem of the target user "How can elderly osteoporosis patients receive traditional Chinese medicine and rehabilitation treatment?";  2.2: The traditional Chinese medicine rehabilitation treatment in the recommended opinions is consistent with the practical scope of the target user;  2.3: Action on practical scope and recommended opinions, such as; Electrotherapy, magnetic therapy, and low-intensity exercise guidance are consistent.  2.4: The direction of recommendations should be consistent with the pros and cons;  2.5: The recommended strength should be balanced against the pros and cons. |  |  | 7 |
|  |  |  |  |  |  | Applicability to Patients/Populations | 3.1: The guidelines include outcomes related to the target patient, such as muscle strength, falls, bone density, etc;  3.2: Mention the impact of electrotherapy, magnetic therapy, and low-intensity exercise on related outcomes. | 3.3：Not mentioned；  3.4：Not mentioned. |  | 4 |
|  |  |  |  |  | Values and Preferences | Values and Preferences of Target Users |  | 4.1: Not mentioned;  4.2: Not mentioned;  4.3: Not mentioned;  4.4: Not mentioned. |  | 1 |
|  |  |  |  |  |  | Values and Preferences of Patients/Populations | 5.4: Describing the benefits of decision-making in improving patient muscle strength, preventing falls, and increasing bone density. | 5.1: Not mentioned;  5.2: Not mentioned;  5.3: Not mentioned. |  | 2 |
|  |  |  |  |  |  | Values and Preferences of Policy/Decision-Makers |  |  | 6.1，6.2，6.3，6.4 | 1 |
|  |  |  |  |  |  | Values and Preferences of Guideline Developers |  | 7.1: Not mentioned;  7.2: Not mentioned;  7.3: Not mentioned. |  | 1 |
|  |  |  |  |  | Implementability | Purpose | 8.1: The recommended opinion is about traditional Chinese medicine rehabilitation treatment for osteoporosis, which is consistent with the goals of the guidelines;  8.2: Describing the impact of adopting recommendations on patients, such as improving muscle strength, preventing falls, and increasing bone density. | 8.2: The expected impact of adopting recommendations on target users, organizations, or systems is not mentioned. |  | 6 |
|  |  |  |  |  |  | Local Application and Adoption | 9.1: Medical institutions with conditions can use multi band spectroscopy, low-frequency pulsed electromagnetic fields, and other treatments;  9.4: Mention the resources required to implement the recommended recommendations, such as multi band spectroscopy and low-frequency pulsed electromagnetic field therapy equipment. | 9.2: Not mentioned;  9.3: Not mentioned. |  | 3 |
|  |  | Triple prevention strategies for elderly osteoporosis | It is recommended to provide primary prevention of osteoporosis for elderly individuals with low and medium risk of fractures, in order to reduce or delay the occurrence of osteoporosis. Preventive measures include health education, lifestyle guidance, risk population screening and risk factor control, fall risk assessment and prevention, calcium and vitamin D supplementation, etc. | 2B | Clinical Applicability | Evidence | 1.2: Multiple pieces of evidence support the same recommendation, indicating that multiple research results are similar, such as the handling of different risk levels;  1.3: The recommendation takes into account the strategic direction of hierarchical prevention, and accurately proposes the population, intervention measures, and benefit outcomes for prevention at all levels;  1.8: Dose response gradients were mentioned, such as the criteria for grading at all levels, the probability of osteoporotic fractures, and the probability of hip fractures. | 1.1: Not mentioned;  1.4: Not mentioned;  1.5: Not mentioned;  1.6: Not mentioned. | 1.7 | 3 |
|  |  |  | It is recommended to provide secondary prevention of osteoporosis for elderly individuals at high risk of fractures, in order to reduce the incidence of fractures. Specific measures include standardizing the use of anti osteoporosis drugs and rehabilitation treatment on the basis of primary prevention | 2B |  | Applicability to Target Users | 2.1: The recommended opinions have addressed the hygiene issues of the target users in the development of a three-level prevention strategy for osteoporosis in the elderly;  2.2: The three-level prevention of osteoporosis in the recommended opinions is consistent with the practical scope of the target users;  2.3: Action on practical scope and recommended opinions, such as; Health guidance, anti osteoporosis medication, surgical treatment, etc.  2.4: The direction of recommendations should be consistent with the pros and cons;  2.5: The recommended strength should be balanced against the pros and cons. |  |  | 7 |
|  |  |  | It is recommended to carry out tertiary prevention of osteoporosis for individuals with extremely high risk of fractures or those with newly developed brittle fractures, in order to reduce the incidence of fractures and fractures, as well as disability and mortality rates. Adopting comprehensive measures including strengthened management, surgical intervention, standardized use of potent anti osteoporosis drugs (such as desuvumab, zoledronic acid, terlipide), and rehabilitation treatment to improve patient quality of life and extend life expectancy. | 2B |  | Applicability to Patients/Populations | 3.1: The guidelines include outcomes related to the target patient, such as bone loss, fractures, re fractures, bone density, quality of life, etc;  3.2: Mention the impact of health education, rehabilitation treatment, etc. on relevant outcomes.  3.4: Different intervention recommendations have been developed for populations with different risk levels. | 3.3: Not mentioned. |  | 6 |
|  |  |  |  |  | Values and Preferences | Values and Preferences of Target Users | 4.3: Differentiated recommendations and suitable for people with different risk levels;  4.4: It is mentioned that individuals with extremely high risk of fractures who have surgical indications should undergo surgery as soon as possible. | 4.1: Not mentioned;  4.2: Not mentioned. |  | 3 |
|  |  |  |  |  |  | Values and Preferences of Patients/Populations | 5.4: Describing the benefits of decision-making in delaying bone loss, preventing fractures and re fractures, improving quality of life, and reducing disability and mortality rates. | 5.1: Not mentioned;  5.2: Not mentioned;  5.3: Not mentioned. |  | 2 |
|  |  |  |  |  |  | Values and Preferences of Policy/Decision-Makers | 6.2: The importance of establishing a three-level prevention and control system for elderly osteoporosis was discussed. | 6.1: Not mentioned;  6.3: Not mentioned;  6.4: Not mentioned. |  | 2 |
|  |  |  |  |  |  | Values and Preferences of Guideline Developers |  | 7.1: Not mentioned;  7.2: Not mentioned;  7.3: Not mentioned. |  | 1 |
|  |  |  |  |  | Implementability | Purpose | 8.1: The recommended suggestion is a graded prevention strategy for osteoporosis, consistent with the goals of the guidelines;  8.2: Describe the impact of adopting recommendations on patients and the population, such as reducing the harm of osteoporosis and its fractures, delaying bone loss, preventing fractures and re fractures, improving quality of life, and reducing disability and mortality rates. | 8.2: The expected impact of adopting recommended opinions on the organization or system is not mentioned. |  | 6 |
|  |  |  |  |  |  | Local Application and Adoption |  | 9.1: Not mentioned;  9.2: Not mentioned;  9.3: Not mentioned;  9.4: Not mentioned. |  | 1 |
| 2 | COA-GTO | Standardized anti osteoporosis treatment after brittle fracture surgery | Carry out health education for perioperative patients | / | Clinical Applicability | Evidence | 1.3: Only mention corresponding beneficial outcomes, such as the risk of falls and fractures, in calcium supplements, vitamin D, and reasonable exercise. | 1.1: Not mentioned;  1.2: Non conformity;  1.3: Incomplete description of exact measures;  1.4: Not mentioned;  1.5: Not mentioned;  1.6: Not mentioned;  1.8: Not mentioned. | 1.7 | 1 |
|  |  |  | Supplementing with sufficient calcium and vitamin D | 2C |  | Applicability to Target Users | 2.1: The recommended opinions have addressed the hygiene issues of target users regarding standardized anti osteoporosis treatment after osteoporotic fractures;  2.2: The health education content for osteoporosis fractures in the recommended opinions is consistent with the practical scope of the target users;  2.3: The scope of practice is consistent with the recommended actions, such as; Health education work, providing health guidance on calcium and vitamin D supplementation, reasonable exercise, smoking cessation, and alcohol restriction.  2.4: Some recommendations align with the pros and cons, such as calcium and vitamin D supplementation, reasonable exercise, smoking cessation, and alcohol restriction. | 2.5: Some recommended opinions do not provide grading intensity, such as health education and reasonable exercise. |  | 5 |
|  |  |  | Exercise reasonably when physical conditions permit | / |  | Applicability to Patients/Populations | 3.1: Including relevant outcomes such as fracture risk, fall risk, bone loss, etc;  3.2: Some recommended opinions mention that implementation can affect patient outcomes, such as sufficient intake of calcium and vitamin D can reduce the risk of fractures, exercise can increase bone synthesis metabolism, and reduce the risk of falls. | 3.3: Not mentioned;  3.4: Not mentioned. |  | 3 |
|  |  |  | It is recommended that all patients who have undergone fragile fracture surgery quit smoking and avoid alcohol abuse | 2B | Values and Preferences | Values and Preferences of Target Users |  | 4.1: Not mentioned;  4.2: Not mentioned;  4.3: Not mentioned;  4.4: Not mentioned. |  | 1 |
|  |  |  |  |  |  | Values and Preferences of Patients/Populations | 5.4: It is beneficial to receive sufficient intake of calcium and vitamin D, exercise reasonably, and reduce the risk of fractures and falls. | 5.1: Not mentioned;  5.2: Not mentioned;  5.3: Not mentioned. |  | 2 |
|  |  |  |  |  |  | Values and Preferences of Policy/Decision-Makers |  | 6.1: Not mentioned;  6.2: Not mentioned;  6.3: Not mentioned;  6.4: Not mentioned. |  | 1 |
|  |  |  |  |  |  | Values and Preferences of Guideline Developers |  | 7.1: Not mentioned;  7.2: Not mentioned;  7.3: Not mentioned. |  | 1 |
|  |  |  |  |  | Implementability | Purpose | 8.1: The recommended opinion is about standardized anti osteoporosis treatment for osteoporotic fractures, which is consistent with the goals of the guidelines;  8.2: Describing the impact of adopting recommended opinions on patients and the population, such as reducing the risk of fractures, reducing the risk of falls, and accelerating bone loss. | 8.2: The expected impact of adopting recommended opinions on the organization or system is not mentioned. |  | 6 |
|  |  |  |  |  |  | Local Application and Adoption |  | 9.1: Not mentioned;  9.2: Not mentioned;  9.3: Not mentioned;  9.4: Not mentioned. |  | 1 |
| 2 | COA | What are the basic measures for the prevention and treatment of osteoporotic fractures? | The basic measures include adjusting lifestyle, preventing falls, and using basic bone health supplements such as calcium supplements and vitamin D, which are the basic guarantees for the maximum effect of anti osteoporosis drugs. | 1A | Clinical Applicability | Evidence | 1.2: A recommendation opinion is supported by multiple research evidence;  1.3: The recommendation takes into account precise intervention measures, population, and benefit outcomes, such as calcium supplements and vitamin D supplementation methods, the population with osteoporotic fractures, and the risk of recurrent fractures. | 1.1: Not mentioned;  1.4: Not mentioned;  1.5: Not mentioned;  1.6: Not mentioned;  1.8: Not mentioned. | 1.7 | 2 |
|  |  | How to prevent postoperative recurrence of osteoporotic fractures? | There is a risk of re fracture during and after surgery for osteoporotic fractures. Regular fracture risk assessment, timely identification of high-risk groups for re fracture, and early standardized systemic intervention can help prevent and treat re fractures. | 2B |  | Applicability to Target Users | 2.1: The recommended opinions have addressed hygiene issues such as "What are the basic measures for preventing and treating osteoporotic fractures?";  2.2: The scope of user practice for preventing osteoporotic fractures and re fractures, as well as perioperative management, is consistent with the target patient;  2.3: The recommended opinions are consistent with the user's actions on preventing recurrent fractures and perioperative management for the target patient;  2.4: Consistent recommendations and trade-offs;  2.5: The recommended strength should be balanced against the pros and cons. |  |  | 7 |
|  |  | How to manage osteoporosis fractures during the perioperative period? | Patients with osteoporotic fractures have multiple comorbidities, high risks of anesthesia and surgery, and multiple postoperative complications. It is crucial for multidisciplinary perioperative management to follow the principle of rapid recovery. | 2B |  | Applicability to Patients/Populations | 3.1: Part includes relevant outcomes, such as promoting bone mineralization, maintaining muscle strength, improving balance, reducing the risk of falls and fractures, etc;  3.2: Some recommendations mention that implementation will affect patient outcomes, such as preventing re fractures and reducing the risk of perioperative complications. | 3.3: Not mentioned;  3.4: Not mentioned. |  | 4 |
|  |  | Rehabilitation strategies for osteoporotic fractures? | The rehabilitation treatment of patients with osteoporotic fractures should not only follow the general rehabilitation rules of fractures, but also consider the characteristics of poor bone quality and slow fracture healing in patients. A personalized comprehensive rehabilitation plan with multiple disciplines and factors should be adopted. | 2B | Values and Preferences | Values and Preferences of Target Users | 4.3: The recommendation mentions that the dosage of calcium supplements varies for different ages, and different rehabilitation exercise methods are adopted for different fracture sites;  4.4: The recommendation mentions the preferred rapid recovery strategy and multidisciplinary team collaboration. | 4.1: Not mentioned;  4.2: Not mentioned. |  | 4 |
|  |  |  |  |  |  | Values and Preferences of Patients/Populations | 5.4: It is beneficial to mention the adoption of recommendation teams to prevent re fractures and reduce the risk of perioperative complications. | 5.1: Not mentioned;  5.2: Not mentioned;  5.3: Not mentioned. |  | 2 |
|  |  |  |  |  |  | Values and Preferences of Policy/Decision-Makers | 6.2: The recommendation mentions that hospitals with conditions should establish elderly orthopedic wards jointly managed by multiple disciplines. | 6.1: Not mentioned;  6.3: Not mentioned;  6.4: Not mentioned. |  | 2 |
|  |  |  |  |  |  | Values and Preferences of Guideline Developers |  | 7.1: Not mentioned;  7.2: Not mentioned;  7.3: Not mentioned. |  | 1 |
|  |  |  |  |  | Implementability | Purpose | 8.1: The recommended opinions are about basic care for osteoporotic fractures, prevention of re fractures, perioperative management, and rehabilitation strategies, consistent with the goals of the guidelines;  8.2: Describe the impact of adopting recommendations on patients and the population, such as preventing re fractures and reducing the risk of perioperative complications. | 8.2: The expected impact of adopting recommended opinions on the organization or system is not mentioned. |  | 6 |
|  |  |  |  |  |  | Local Application and Adoption | 9.1: The recommendation mentions that the principle of rapid recovery needs to be implemented under multidisciplinary management. | 9.2: Not mentioned;  9.3: Not mentioned;  9.4: Not mentioned. |  | 2 |
| 3 | CRMA | What functions do patients with osteoporosis need to assess and how do they assess them? | It is recommended to evaluate the five physical functions of osteoporosis patients, including pain, joint range of motion, muscle strength, balance function, and psychological state. It is recommended to use visual analog scoring for pain assessment; It is recommended to use a protractor to evaluate the range of joint movement; It is recommended to use the bare handed muscle strength test method for muscle strength assessment; The evaluation of balance function is recommended to use the Berg Balance Scale; It is recommended to use the Hamilton Anxiety Scale and/or the Hamilton Depression Scale for psychological state assessment | 1D | Clinical Applicability | Evidence | 1.1: The guideline team evaluated the risk of bias in the inclusion study;  1.2: The results are consistent;  1.3: The guidelines consider the direction of clinical evidence and provide precise intervention measures, population, and benefit outcomes, such as low-frequency pulsed electromagnetic field therapy, aerobic exercise, etc;  1.4: The recommendation describes the confidence interval of the evidence;  1.5: The guidelines describe the advantages of recommended opinions, such as the potential benefits of Tai Chi in improving patient bone density, osteocalcin levels, and alleviating osteoporotic pain. The disadvantages include the small sample size of time or frequency dependent effect value studies on pulsed electromagnetic field therapy;  1.8: The recommendation mentions dose response gradients regarding the duration of sunlight exposure. | 1.6: Not mentioned. | 1.7 | 6 |
|  |  | How to evaluate the daily living activities of patients with osteoporosis? | It is recommended to use the improved Barthel index rating scale to assess the daily living activities of patients. | 1C |  | Applicability to Target Users | 2.1: Recommended suggestions have addressed hygiene issues related to osteoporosis rehabilitation;  2.2: The scope of user practice for functional assessment, exercise, training, and health education of osteoporosis is consistent with the target patient;  2.3: The recommended opinions are consistent with the user's functional evaluation, rehabilitation training, home environment renovation, and other actions for the target patient;  2.4: Consistent recommendations and trade-offs;  2.5: The recommended strength should be balanced against the pros and cons. |  |  | 7 |
|  |  | How to evaluate the quality of life of patients with osteoporosis? | Recommend using the Chinese Osteoporosis Brief Quality of Life Scale to assess the quality of life of patients. | 1C |  | Applicability to Patients/Populations | 3.1: Including relevant outcomes such as pain, quality of life, muscle strength, etc;  3.2: Some recommendations mention that implementation will affect patient outcomes. For example, aerobic exercise recommendations mention that appropriate exercise can not only generate mechanical stimulation to promote bone formation, but also regulate the body's endocrine system and increase estrogen levels, thereby promoting bone formation to a certain extent, effectively preventing bone loss and playing a role in preventing and treating osteoporosis;  3.4: For aerobic exercise, different exercise plans are recommended for individuals who exercise regularly and engage in irregular physical activities. | 3.3: Not mentioned. |  | 5 |
|  |  | What is the therapeutic effect of low-frequency pulsed electromagnetic fields on osteoporosis? | Low frequency pulsed electromagnetic fields can improve the pain and quality of life of patients with osteoporosis, and can serve as auxiliary rehabilitation measures for osteoporosis. | 2D | Values and Preferences | Values and Preferences of Target Users | 4.1: Discussions were conducted on user values and preferences. For example, in the functional assessment of osteoporosis patients, it was mentioned that most rehabilitation physicians and therapists believe that balance function is extremely important for osteoporosis patients;  4.3: There is a relevant description, such as home environment transformation, which is personalized transformation based on the patient's family economic conditions and actual family environment, and has flexibility. | 4.2: Not mentioned;  4.4: Not mentioned. |  | 4 |
|  |  | What is the impact of aerobic exercise on patients with osteoporosis? | Recommend aerobic exercise for patients with osteoporosis. For elderly people, low-intensity daily activities and sports are recommended; For patients with chronic lower back pain, carry out stretching exercises that do not increase spinal load and forward bending load. | 1D |  | Values and Preferences of Patients/Populations | 5.1: Conduct a patient preference survey on the preliminary recommendations of the guidelines for 100 patients with osteoporosis;  5.2: The recommendation takes into account the factors that influence the patient's acceptance of the opinion, such as difficulty in movement, severe pain, fear of exercise, etc., which affect the patient's acceptance of aerobic exercise recommendations;  5.3: The guidelines describe how to form recommendations based on the patient's values and preferences;  5.4: Describe the corresponding benefits, such as the ability of progressive resistance training to increase muscle cross-sectional area, muscle fiber count, etc. |  |  | 7 |
|  |  | What is the therapeutic effect of resistance training on patients with osteoporosis? | It is recommended to guide patients with osteoporosis to undergo resistance training, and the specific training intensity should be determined by the rehabilitation therapist based on the patient's assessment of the condition. | 2D |  | Values and Preferences of Policy/Decision-Makers |  | 6.1: Not mentioned;  6.3: Not mentioned;  6.4: Not mentioned. |  | 1 |
|  |  | The impact of whole-body vibration training on patients with osteoporosis? | Whole body vibration training can serve as a therapeutic approach to improve bone density, exercise ability, and related functional parameters in patients with osteoporosis | 2D |  | Values and Preferences of Guideline Developers | 7.4: The integration method of values and preferences was mentioned, such as 23 consensus experts balancing the pros and cons of recommendation opinions and resource utilization based on the quality of evidence presented by the development team. At the same time, combining the patient's wishes and values, the final recommendation opinion was formed through an improved Delphi method. | 7.1: Not mentioned;  7.2: Not mentioned. |  | 3 |
|  |  | Can balance training effectively reduce the risk of falls in patients with osteoporosis? | Recommend balance training for patients with osteoporosis to improve balance ability, prevent falls and fractures | 1C | Implementability | Purpose | 8.1: The recommended opinions are related to rehabilitation strategies such as functional assessment, exercise, training, and health education, which are consistent with the goals of the guidelines;  8.2: Described the impact of adopting recommendations on patients and the population, such as improving bone density, exercise ability, and related functional parameters through whole-body vibration training. | 8.2: The expected impact of adopting recommended opinions on the organization or system is not mentioned. |  | 6 |
|  |  | What is the impact of using assistive devices on patients with osteoporosis? | It is recommended to use crutches or hip protectors for patients with a higher risk of falling; Use of spinal stents for patients with acute or subacute osteoporotic vertebral fractures | 2D |  | Local Application and Adoption | 9.3: The guide mentions the methods of dissemination and promotion;  9.4: The guidelines mention both favorable and unfavorable factors in the implementation of the guidelines. | 9.1: Not mentioned;  9.2: Not mentioned. |  | 4 |
|  |  | What is the impact of home living environment renovation on patients with osteoporosis? | Suggest improving the home environment for osteoporosis patients to prevent falls | 2D |  |  |  |  |  |  |
|  |  | How long is sunshine safe and effective for patients with osteoporosis? | It is recommended that patients with osteoporosis engage in reasonable outdoor activities, ensuring sufficient sunlight exposure for a duration of more than 30 minutes per day. However, the longer the exposure time, the better the effect | 1C |  |  |  |  |  |  |
|  |  | How to provide dietary intervention for patients with osteoporosis? | It is recommended to implement early nutritional intervention for patients with osteoporosis, adjust their dietary structure, consume high-quality protein and high calcium diets, limit the intake of alcohol, coffee, and carbonated drinks, quit smoking, and try to avoid or minimize the use of drugs that affect bone metabolism | 2D |  |  |  |  |  |  |
|  |  | What health education programs have good rehabilitation effects on patients with osteoporosis? | Recommend health promotion and education for patients with osteoporosis, including informing them of the risk factors, hazards, medication knowledge, and dietary structure of osteoporosis | 1B |  |  |  |  |  |  |
|  |  | How to assess the risk of falls in patients with osteoporosis? | It is recommended to use the MORSE Fall Assessment Scale to assess the risk of falls in patients, while also assessing the patient's living environment | 1C |  |  |  |  |  |  |
|  |  | How to assess the risk of fractures in patients with osteoporosis? | Recommend using fracture risk prediction tools to assess the fracture risk of patients | 1A |  |  |  |  |  |  |
|  |  | Does psychological intervention affect the functional status, social relationships, and emotional health of patients with osteoporosis? | Recommend targeted psychological intervention for patients with osteoporosis to help alleviate anxiety, face the disease with a good psychological state, and improve their quality of life | 1C |  |  |  |  |  |  |
|  |  | What is the impact of traditional Chinese medicine techniques (Tai Chi, Ba Duan Jin, and Wu Qin Xi) on patients with osteoporosis? | It is recommended that patients with osteoporosis undergo Tai Chi, Eight Section Brocade, and Five Animal Play exercises, which should be determined based on the patient's assessment of their condition and interests | 1C |  |  |  |  |  |  |
| 4 | CACM | Functional exercise | Reasonable selection of local or systemic functional exercise methods during fracture immobilization can promote limb function recovery | 1B | Clinical Applicability | Evidence | 1.2: Multiple studies support the same evidence;  1.3: Considering the research direction of clinical issues, only mentioning the outcome of benefits;  1.5: Mention the benefits and some drawbacks; Traditional techniques have the advantages of relieving pain, increasing bone density, improving patient quality of life, and avoiding recurrent fractures. | 1.1: Not mentioned;  1.3: Considering the research direction of clinical issues, without proposing specific intervention measures and populations;  1.4: Not mentioned;  1.6: Not mentioned;  1.8: Not mentioned. | 1.7 | 3 |
|  |  | Traditional martial arts therapy | It is recommended that patients with osteoporotic fractures choose traditional techniques such as Eight Section Brocade, Five Animal Play, and Tai Chi for exercise during the rehabilitation period | 1C |  | Applicability to Target Users | 2.1: Partially solved clinical issues related to target users, such as functional exercise and the selection of traditional techniques;  2.2: The scope of practice is consistent with the target audience;  2.3: The scope of practice is consistent with the recommended actions;  2.4: Have consistency;  2.5: Consistent. | 2.1: Without a precise description of the specific implementation measures for functional exercise and traditional techniques, it cannot fully solve the clinical problems of the target users. |  | 5 |
|  |  |  |  |  |  | Applicability to Patients/Populations | 3.1: Including relevant outcomes such as limb function, pain, bone density, quality of life, recurrent fractures, etc;  3.2: The recommendation mentions that implementation will affect patient outcomes, such as functional exercise promoting limb function recovery. | 3.3: Not mentioned;  3.4: Not mentioned. |  | 4 |
|  |  |  |  |  | Values and Preferences | Values and Preferences of Target Users | 4.3: It is mentioned that patients choose improved or simplified versions of the exercises based on their own situation, and the amount of exercise varies from person to person. | 4.1: Not mentioned;  4.2: Not mentioned;  4.4: Not mentioned. |  | 3 |
|  |  |  |  |  |  | Values and Preferences of Patients/Populations | 5.1: The strength of recommendations mentioned in the guidelines takes into account the patient's values and willingness;  5.3: The guidelines take into account the patient's values and preferences to develop recommendations, such as traditional methods that consider the patient's own situation and vary from person to person;  5.4: The guide describes the benefits of implementing recommendations, such as functional exercise promoting limb function recovery. | 5.2: Not mentioned. |  | 5 |
|  |  |  |  |  |  | Values and Preferences of Policy/Decision-Makers | 6.2: The guidelines describe the development of recommendations that take into account medical insurance costs. | 6.1: Not mentioned;  6.3: Not mentioned;  6.4: Not mentioned. |  | 2 |
|  |  |  |  |  |  | Values and Preferences of Guideline Developers |  | 7.1: Not mentioned;  7.2: Not mentioned;  7.3: Not mentioned. |  | 1 |
|  |  |  |  |  | Implementability | Purpose | 8.1: The recommended opinion is about the strategies for traditional Chinese medicine functional exercise and traditional martial arts for patients with osteoporosis, which is consistent with the goals of the guidelines;  8.2: Describe the impact of adopting recommended opinions on patients and the population, such as the role of functional exercise in promoting limb function recovery. | 8.2: The expected impact of adopting recommended opinions on the organization or system is not mentioned. |  | 6 |
|  |  |  |  |  |  | Local Application and Adoption | 9.2: The strength of recommendations mentioned in the guidelines takes into account the differences between the required clinical conditions and practice, geographical compliance, and medical insurance costs. | 9.1: Not mentioned;  9.3: Not mentioned;  9.4: Not mentioned. |  | 3 |
| 6 | Qiu ML et al | / | Low body weight is one of the risk factors for OP occurrence | 1C | Clinical Applicability | Evidence | 1.1: The guidelines have conducted a risk assessment of bias on the included evidence;  1.2: The same recommendation opinion is supported by multiple pieces of evidence;  1.3: The guidelines consider the directionality of clinical issues, such as appropriate exercise, mention specific exercise plans and corresponding benefit outcomes;  1.5: The guide mentions the advantages and disadvantages of recommended opinions, such as participating in patient associations can enhance doctor-patient communication, improve disease management skills and diagnosis and treatment levels, etc;  1.8: The guidelines mention dose-response gradients for alcohol consumption and the risk of OP disease. | 1.4: Not mentioned;  1.6: Not mentioned. | 1.7 | 5 |
|  |  |  | Suggest OP patients to quit smoking | 1C |  | Applicability to Target Users | 2.1: Solved clinical issues related to target users, such as risk factors for OP, appropriate exercise, calcium supplements, and vitamin D supplementation;  2.2: The scope of practice is consistent with the target audience;  2.3: The scope of practice is consistent with the recommended actions;  2.4: Have consistency;  2.5: Have consistency. |  |  | 7 |
|  |  |  | Excessive or long-term alcohol consumption is associated with the occurrence of osteoporosis and the risk of fractures | 1C |  | Applicability to Patients/Populations | 3.1: The guidelines include outcomes related to the target patient, such as OP disease risk, bone density, risk of falls and fractures, etc;  3.2: Mention the impact of appropriate exercise, patient associations, etc. on relevant outcomes;  3.4: Aerobic exercise with weight-bearing is more suitable for patients with severe OP and those in the recovery period of fractures. | 3.3: Not mentioned. |  | 6 |
|  |  |  | Bone density examination can be used for OP warning, diagnosis, and drug efficacy evaluation | 1B | Values and Preferences | Values and Preferences of Target Users | 4.3: Aerobic exercise with weight-bearing is more suitable for patients with severe OP and those in the recovery period of fractures. | 4.1: Not mentioned;  4.2: Not mentioned;  4.4: Not mentioned. |  | 2 |
|  |  |  | Appropriate exercise for OP patients is beneficial for protecting bones | 1B |  | Values and Preferences of Patients/Populations | 5.1: The guidelines mention that the formation of recommended opinions takes into account the patient's values and preferences;  5.4: Mention the benefits of appropriate exercise, patient gatherings, etc. for patients. | 5.2: Not mentioned;  5.3: Not mentioned. |  | 3 |
|  |  |  | Supplementing calcium and vitamin D simultaneously is a fundamental measure for preventing and treating osteoporosis | 1B |  | Values and Preferences of Policy/Decision-Makers | 6.3: The guidelines mention that the formation of recommended opinions takes into account the cost of intervention measures. | 6.1: Not mentioned;  6.3: Not mentioned;  6.4: Not mentioned. |  | 2 |
|  |  |  | Suggest osteoporosis patients to participate in patient associations | 1B |  | Values and Preferences of Guideline Developers |  | 7.1: Not mentioned;  7.2: Not mentioned;  7.3: Not mentioned. |  | 1 |
|  |  |  |  |  | Implementability | Purpose | 8.1: The recommended opinions are related to risk factor intervention, appropriate exercise, calcium and vitamin D supplementation, and patient association intervention measures for osteoporosis patients, which are consistent with the goals of the guidelines;  8.2: Describing the impact of adopting recommended opinions on patients and the population, such as the effects of appropriate exercise on maintaining and improving bone density, reducing the risk of falls and fractures, etc. | 8.2: The expected impact of adopting recommended opinions on the organization or system is not mentioned. |  | 6 |
|  |  |  |  |  |  | Local Application and Adoption | 9.1: When it comes to calcium supplements and vitamin D supplementation, the recommendation mentions that vitamin D deficiency is commonly present in the Chinese population. | 9.2: Not mentioned;  9.3: Not mentioned;  9.4: Not mentioned. |  | 2 |
